# The effector repertoire of enteropathogenic *E. coli*: ganging up on the host cell

**DOI:** 10.1016/j.mib.2008.11.006

**Published:** 2009-02

**Authors:** Paul Dean, Brendan Kenny

**Affiliations:** Institute for Cell and Molecular Biosciences, Medical School, Newcastle University, Catherine Cookson Building, Framlington Place, Newcastle upon Tyne NE2 4HH, UK

## Abstract

Diarrhoeal disease caused by enteropathogenic *E. coli* (EPEC) is dependent on a delivery system that injects numerous bacterial ‘effector’ proteins directly into host cells. The best-described EPEC effectors are encoded together on the locus of enterocyte effacement (LEE) pathogenicity island and display high levels of multifunctionality and cooperativity within the host cell. More recently, effectors encoded outside the LEE (non-LEE effectors) have been discovered and their functions are beginning to be uncovered. The recent completion of the EPEC genome sequence suggests its effector repertoire consists of at least 21 effector proteins. Here, we describe the genomic location of effectors and discuss recent advances made on effector cellular function as well as their role in the infection process.

## Introduction

Enteropathogenic *E. coli* (EPEC) is a human pathogen of the small intestine that causes severe watery diarrhoea, particularly among infants in developing countries [1]. EPEC is a member of a closely related family of pathogens that induce characteristic attaching and effacing (A/E) lesions on intestinal epithelial cells in humans (EPEC and EHEC — enterohaemorrhagic *E. coli*), ruminants (EHEC) and small animals including mice (*Citrobacter rodentium*) [2]. Hallmarks of EPEC disease are loss (effacement) of absorptive microvilli, induction of actin-rich pedestals underneath adherent bacteria, rapid watery diarrhoea, inhibition of nutrient/water transporter function, mitochondrial dysfunction, a weak inflammatory response and tight junction (TJ) disruption (see [1,3]). Upon initial contact with intestinal enterocytes, mediated in part by the bundle forming pilus (BFP), EPEC rapidly cause the effacement of microvilli and induce localised actin polymerisation that gives rise to a pedestal beneath the bacteria, to which the bacteria intimately attach. Mitochondrial dysfunction and disruption of nutrient transporters are also early events, whilst the disruption of TJ is only apparent later on during the infection process. EPEC is considered a non-invasive pathogen and relies upon on a type three secretion system (T3SS) to deliver effector proteins directly into host cells which subvert a myriad of host cellular functions, ultimately leading to disease [3]. The first EPEC effectors to be discovered are all encoded on a large genomic pathogenicity island called the locus of enterocyte effacement (LEE), which also carries EPEC's only T3SS, with seven LEE effectors identified to date. More recently, effectors encoded outside the LEE region have been found in all A/E pathogens [4,5] which utilise the LEE T3SS for delivery into host cells and recent functional studies on these ‘non-LEE’ effectors have started to ascribe cellular functions to these proteins. The completion of the genome sequence of the prototypical EPEC strain E2348/69 (herein termed EPEC; www.sanger.ac.uk/Projects/Escherichia_Shigella) has confirmed the presence (or absence) of many non-LEE effector genes [6^•^].

EPEC also possess a type two secretion system (T2SS), although little is known about its role in virulence or its protein substrates [6^•^], and a type five secretion system (T5SS) which includes the enterotoxin EspC and other putative autotransporters [7,8^••^]. Interestingly, the entry of EspC into the host cells has been shown to be dependent on T3SS [8^••^] and it is likely that other autotransporters may also depend on the T3SS. In this review we will focus on the repertoire of type three secreted EPEC effector proteins and the important advances made on effector functions over the past few years. We redirect the reader to other reviews for information on other virulence factors and general mechanisms of EPEC pathogenesis [1,3,7].

## EPEC effector nomenclature

The naming of EPEC effectors has been based on three approaches and may be somewhat confusing to the lay reader. Traditionally, the term ‘Esp-’ was used to denote *E*PEC *s*ecreted *p*rotein and now includes the LEE effectors EspB/F/G/H. However, EspA and EspD, which have not been reported to possess effector activity, and the autotransporter EspC, also fall into this category, making effector designation a little ambiguous. A different approach was to name effectors to reflect their reported function, thus the LEE effectors Translocated Intimin receptor (Tir) and Mitochondrial-associated protein (Map) fall into this group. Finally, the term *n*on-*L*EE *e*ncoded (Nle-) was coined by Deng *et al.* following their discovery of several non-LEE effectors in *Citrobacter* [4]. However, this designation has not been adopted for all non-LEE effectors — such as the non-LEE located EspG2/Orf3 [9], EspI/NleA [10], cycle inhibiting factor (Cif) [11] and a set of recently discovered non-LEE effectors in EHEC, which have all been given the prefix ‘Esp-’ [5], of which EspJ and EspL are encoded in EPEC (Table 1).

## The LEE effectors — highly interdependent and multifunctional

Historically, the LEE effectors were the first to be identified in EPEC and to date a total of seven LEE-encoded proteins delivered into the host cell have been discovered, namely Tir, Map, EspF, EspG, EspZ (previously SepZ), EspH and EspB (which is also a translocator and essential for the delivery of effectors into the host cell) (see Table 1 and [3]). Also present on the LEE is the outer membrane protein Intimin which, whilst not delivered into host cells, causes numerous host cell responses directly through its bacterial-encoded receptor Tir or through various host cell receptors [1,3]. All the LEE effectors, except EspZ [12], have proven deleterious effects on the host cell (Table 1 and see [3]) and it is likely that EspZ has important effector functions as it is delivered early in the infection process and at similarly high levels as the essential virulence determinant, Tir [13]. The importance of the LEE effectors in the disease process is unclear but animal infections using *Citrobacter* and EHEC (as EPEC lacks a suitable animal model) indicate that Tir is essential, linked to its role in bacterial attachment, whilst the other LEE effectors have a smaller but additive contribution to virulence [2,10,14,15].

**Figure 1:**
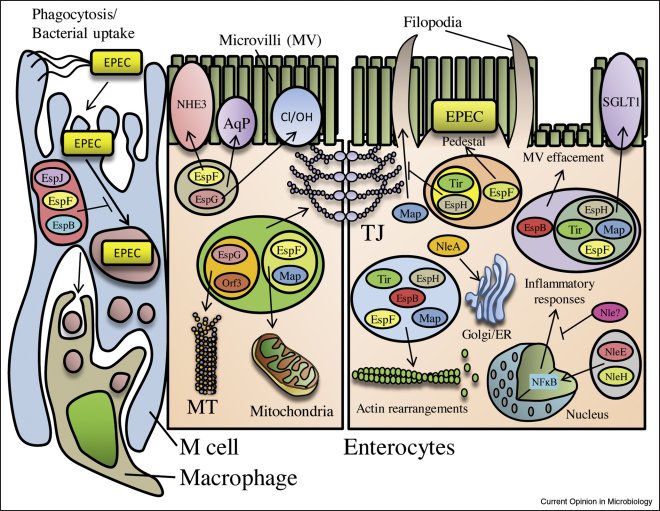
The complexity of EPEC effector function. The multifunctional and overlapping properties of the EPEC effectors are depicted here by grouping effector functions together. Three effectors have anti-phagocytic activities (shown here using the phagocytic-like gut-associated antigen presenting M-cells) whilst at least five effectors act on microvilli and four inhibit SGLT-1 and other transporter activity, four disrupt tight junctions and three are involved in pedestal and filopodia formation. At least three Nle effectors are also involved in inflammatory pathways. Microtubule and Golgi/ER disruption appears to be specific to EspG/Orf3 and NleA, respectively. Also shown are effectors which have known actin-modulating properties. TJ, tight junctions; MT, microtubules; AqP, aquaporins; NHE3, sodium hydrogen exchanger; Cl/OH, Cl^−^/OH^−^ transporter; SGLT-1, sodium glucose cotransporter-1; ER, endoplasmic reticulum; MV, microvilli.

The functions of the LEE effectors are highly varied and Table 1 gives an up-to-date and comprehensive list of reported effector functions. An emerging theme for the LEE effectors, consistent with findings in other T3SS-pathogens, is their multiple and overlapping functions (termed functional redundancy) and their interdependence and cooperativity in subverting host cell activities (Table 1 and Figure 1). For example, Map and EspF synergise [16] whilst EspG and EspG2 function redundantly [17] in the disruption of epithelial TJ, with the Map/EspF TJ-disrupting activity proven *in vivo* [14,18]. Tir is essential for actin-pedestal formation following binding to Intimin but is also involved in TJ disruption, independent and dependent of Intimin (Dean and Kenny, unpublished). Tir also downregulates Map-induced filopodia formation (see [3]) and coordinates with EspF, Intimin and Map to cause microvilli effacement [19^•^]. Indeed, Knutton and colleagues have also reported overlapping roles for LEE effectors in microvilli effacement *ex vivo*, using human intestinal material [20]. In addition, Map and EspF both target mitochondria to alter organelle shape and cause dysfunction — an activity that occurs *in vivo* and proven to be important in disease [14,15]. Such effector cooperativity appears to be just the ‘tip of the iceberg’ as a systematic genetic study in which the LEE effectors were deleted in many different combinations eludes to an unprecedented level of functional cooperativity between effectors (Kenny *et al.*, unpublished). To this end, all the major reported hallmarks of EPEC disease can be attributed to the cooperative efforts of the LEE effectors (Figure 1).

**Figure 2:**
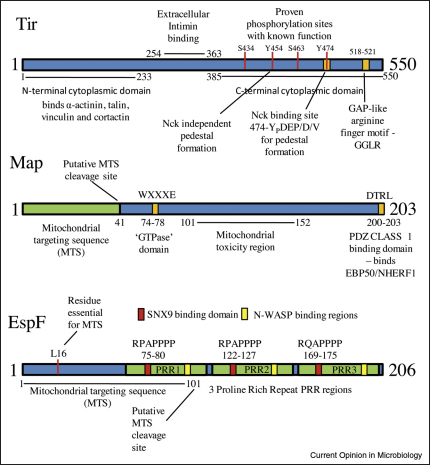
The modular nature of multifunctional LEE effector proteins. Tir, Map and EspF are the best-studied EPEC effectors and have been implicated in subverting multiple cellular processes. These proteins possess many eukaryotic-like motifs with many being assigned to elicit specific host cellular responses. Only those motifs/domains with proven and documented functions within the host cell are shown whilst chaperone binding sites or the N-terminal bacterial secretion and translocation signals are not shown. Other EPEC effectors are not shown because of the paucity of information regarding their functional domains. See text for abbreviations.

In addition to their cooperative nature, the LEE effectors are strikingly multifunctional (Table 1) binding a large number of eukaryotic proteins and targeting various host cell compartments (Table 1 and Figure 1). This is best exemplified by EspF which localises to multiple cellular compartments (including cytoplasm, mitochondria, apical and lateral membranes) and interacts with at least 12 reported host proteins, with its delivery linked to mitochondrial dysfunction, microvilli effacement, TJ disruption, apoptosis, epithelial transporter inhibition, anti-phagocytosis, membrane remodelling and actin-pedestal maturation [15,16,18–22,26,51–53]. Like other LEE effectors, the modular construction of EspF facilitates its multifunctional behaviour (Figure 2), with specific motifs inducing distinct cellular responses, such as an N-terminal mitochondrial targeting sequence (MTS) linked to mitochondria dysfunction and apoptosis [15,21], whilst proline-rich repeats, that include src homology 3 (SH3)-binding domains, recruit sorting nexin 9 (SNX9) causing membrane remodelling ([22]; Table 1 and Figure 2). EspF, like its EHEC homologues EspF and EspF_U_/Tccp, recruits N-WASP (a key regulator of actin polymerisation), with studies on EspF_U_ revealing it specifically activates N-WASP by mimicking an internal regulatory element [23,24]. Finally, although EspF plays an essential role in anti-phagocytosis, EspB–myosin interaction has recently been reported to inhibit both phagocytosis and microvilli effacement [25^•^]. However, as both of these bacterial processes can occur with EspB/EspF-positive but not EspB-positive/EspF-negative strains [19^•^,26]; this suggests that EspB is neither solely responsible nor sufficient.

Effector multifunctionality is further demonstrated by Map and Tir which like EspF, possess motifs that mediate a broad array of functions ([3]; Table 1 and Figure 2). All of Tir's reported activities depend on its extracellular domain (Figure 2) binding to Intimin, following Tir insertion into the host plasma membrane. Tir's N-terminal and C-terminal domains remain intracellular and interact with numerous signalling, adapter and cytoskeletal proteins with Tir function dependent on its phosphorylation sites, a GTPase activating protein (GAP)-like motif and a polyproline region (Figure 2) [27–29]. Interestingly, although tyrosine phosphorylation of Tir is essential for actin-pedestal formation in immortalised cell lines, human biopsy material has revealed that this crucial event is independent of tyrosine phosphorylation *ex vivo*, suggesting care should be taken when using cell lines to elucidate effector function [30^••^]. Map on the other hand is reported to mimic the active form of Cdc42 — a small GTPase — to induce filopodia formation [31], in contrast to an earlier report demonstrating a dependence on Cdc-42 itself [32]. The filopodia-inducing functions of Map depend on an invariant WxxxE motif and a C-terminal class 1 PDZ-binding domain that binds Ezrin-binding protein 50 [31,33] to presumably direct or retain Map to the plasma membrane. Whether or not Map is a Cdc42 mimic is open to debate as other effectors of the WxxxE family appear to require GTPases to elicit their cellular responses [32,34^••^] and the controversy surrounding the WxxxE effector family has been eloquently discussed in a recent review which provides a compelling argument to suggest these effectors may not be GTPase mimics after all [34^••^]. Finally, Map possesses an N-terminal MTS and targets the mitochondria where it is imported via the classical TOM/Hsp70 import system and causes mitochondrial dysfunction [35] (see Figures 1 and 2).

## EPEC non-LEE effectors and non-LEE pathogenicity islands

**Figure 3:**
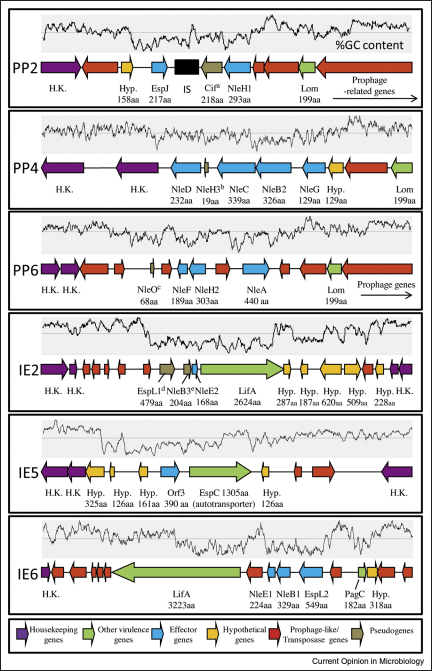
The six identified non-LEE effector encoding pathogenicity islands of EPEC E2348/69. Predicted effector genes were identified by mining the EPEC genome using over 400 known/predicted effector sequences. The identified effectors and genomic islands support the genome sequence published data (see text), from which the genomic island names were obtained. Only those genomic regions encoding the effectors and with low %GC content (graph above each island) are shown with most prophage-related genes surrounding these regions omitted. Genes and strand direction are shown by individual arrows which are drawn to scale within each island and colour coded (see inset). Multiple copies of genes are numbered according to close homologues in EHEC as explained in the legend to Table 1. Pseudogene key: (a) Cif; C-terminally truncated protein not produced or secreted in this EPEC strain [48]; (b) NleH3; C-terminal truncated; (c) NleO; no start codon; (d) EspL1; stop codon in middle of gene; (e) NleB3; N-terminal truncated.

Whilst mining the EHEC (O157:H7 Sakai strain) genome sequence with over 200 known/predicted T3SS-dependent effector proteins, Pallen and colleagues identified 49 putative effectors [5]. At least 39 of these predicted proteins, of which many are homologues, were confirmed as secreted effectors in EHEC and include NleA-H (noting 12 NleG homologues) and newly described EspJ-O, EspR and EspV-Y effectors [5]. The recent completion of the EPEC genome sequence (strain E2348/69) enabled a similar ‘effector mining’ approach using an expanded list of over 400 known/predicted effector sequences and identified only 21 putative effectors (Dean and Kenny, unpublished; Table 1 and Figure 3). Thus, as recently reported [6^•^], EPEC appears to have a much smaller non-LEE effector repertoire than EHEC, encoding NleA-H (two homologues of B, E, H and only 1 of NleG), EspJ and EspL2, Orf3 (EspG2; which is the only EPEC effector so far identified that is not present in EHEC) and pseudogenes for NleH, EspO, NleB, EspL and Cif (Table 1 and Figure 3). Thus, whilst the LEE effector repertoire is well conserved, the set of non-LEE effectors is apparently flexible as EPEC strains B171-8 and E22 (rabbit-EPEC) possess 28 and 40 effectors, respectively, compared to 21 for the prototypical strain [6^•^].

The non-LEE effector genes are clustered in six pathogenicity islands (Figure 3) scattered throughout the genome, usually with a low G + C% content in regions corresponding to effector genes. Interestingly, the non-LEE effector genes are surrounded by phage-related and/or transposase-like genes implying that, like most pathogenicity islands, they were acquired through horizontal acquisition [6^•^]. Whilst EPEC E2348/69 carries at least 21 effector-encoding genes, it is not known whether they are all expressed but undoubtedly other effectors remain to be identified. Indeed, there are many hypothetical genes in the non-LEE islands that are likely candidates as effectors because of their low %GC content and close proximity to known effector genes (Figure 3).

Because of the more recent discovery of non-LEE effectors, relatively little is known about their cellular function (summarised in Table 1), but NleA is reported to inhibit protein secretion [36^•^], EspJ inhibits phagocytosis [37], whilst NleE [38] and NleH [39] activate innate immune responses. Studies with the mouse model suggest that EspJ, NleB, NleE, NleF and NleH play a role in colonisation and full virulence [40–43] whilst NleC and D have no detectable function [40,41]. Although NleA (also called EspI) was reported to be a key virulence factor [44], another study using the same mouse model, reported that it only contributes to full virulence — as with most other effectors [10]. Interestingly, despite NleE and NleH reportedly inducing innate immune responses, EPEC T3SS-dependent function has been demonstrated to inhibit, not activate, such responses in small intestinal cells and mouse studies [45^•^,46]. Importantly, the reported inhibitory mechanism was not dependent on LEE effectors, implicating non-LEE effectors in the process [45^•^] although a LEE effector was recently identified that inhibits NF-κB activation (Kenny *et al.*, unpublished) — revealing yet another overlapping role for LEE and non-LEE effectors. Therefore, a discrepancy exists as NleE and NleH appear to induce inflammatory responses, whilst EPEC's overall effect on host/host cells is anti-inflammatory. It is possible that EPEC transiently induces pro-inflammatory responses but rapidly inactivates this response mechanism before the disruption of TJs, that is associated with onset of inflammatory cascades [45^•^]. Thus, like the activities of many other EPEC effectors, a delicate balance likely exists between pro-inflammatory and anti-inflammatory signalling mechanisms [47].

## Conclusions

‘Multifunctional, cooperative and redundant’ are three overriding themes that describe EPEC effector behaviour and are becoming increasingly accepted for various T3SS-pathogen effectors. Interestingly, the LEE effectors appear to subvert many ‘core’ epithelial cell processes and consequently, all of the major EPEC disease-related hallmarks have been attributed to the LEE effectors, suggesting the non-LEE effectors may function mainly as accessory/efficiency factors. However, it is worth pointing out that individual non-LEE effectors are more highly conserved between A/E pathogens than LEE effectors [6^•^], possibly reflecting the targeting of well conserved processes, whilst LEE-effector variability may reflect different host ranges. The high level of functional interdependence between the LEE effectors possibly reflects their continued co-evolution within the LEE pathogenicity island and this sets an exciting precedent that other effectors co-inherited together may display similarly complex levels of interplay. A future challenge will be to define the contribution of all effectors in each disease process by identifying the effector domains and motifs responsible. Only then the contribution of the particular effector function to the disease process can be assessed in animal models. Nonetheless the expanded repertoire of effectors provides fascinating opportunities to understand how pathogens subvert cellular processes and increase our understanding of vital eukaryotic processes.

## References and recommended reading

Papers of particular interest, published within the period of review, have been highlighted as:

• of special interest
•• of outstanding interest

## Figures and Tables

**Table 1:** The effector repertoire of EPEC 2348/69 and known functional characteristics of effector proteins.

EPEC effector	Island location	Cellular/physiological functions	Subcellular target sites	Proposed host partners	Functional motifs	Homologue family
Tir	LEE	Actin polymerisation	Plasma membrane	IQGAP1	SH3-binding domains	None known
		TJ disruption^a^	Cytoplasm	14-3-3tau	GAP motif	
		Cell detachment		Nck	Phos. sites	
		Microvilli effacement		α-Actinin		
		SGLT-1 inactivation		Talin		
		PLCγ phosphorylation		Cortactin		
		Regulating effector activity^a^		Vinculin		
		Invasion non-polarised cells		Cytokeratin 18		

Map	LEE	TJ disruption	Mitochondria	EBP50/	PDZ1-binding domain	IpgB2
		Filopodia formation	Actin^a^	NHERF1	MTS	
		Mitochondrial dysfunction	Cytoplasm		WxxxE	
		Microvilli effacement				
		SGLT-1 inactivation				
		Invasion non-polarised cells				

EspB	LEE	Anti-phagocytosis	Cytoplasm	Antitrypsin		YopD
		Microvilli effacement	Plasma membrane	α-Catenin		
		Actin disruption		Myosin		
		Pore formation				

EspF	LEE	Apoptosis	Mitochondria, cytoplasm	ABCF2	PRR,	None known except other EspF variants such as EspF(U)
		TJ disruption	Apical and lateral membranes	Actin	SH3	
		Microvilli effacement	TJ region^a^	ZO-1/ZO-2	N-WASP and SNX9 binding domains	
		Microvilli elongation		Profilin	MTS	
		SGLT-1 inactivation		Arp2/3		
		Mitochondrial dysfunction		Cytokeratin18		
		Pedestal maturation		Sorting nexin 9		
		Inhibition of NHE3 activity		N-WASP		
		Membrane remodelling		14-3-3		
		Aquaporin redistribution		Mito protein^b^		
		N-WASP activation				

EspH	LEE	Modulating actin dynamics	Pedestals			None known
		Cytoskeleton disruption	Plasma membrane			

EspZ	LEE	Unknown	Pedestals			None known

EspG	LEE	Microtubule disruption	Microtubule colocalisation	Tubulin		VirA
		TJ disruption				
		Paracellular permeability				
		Aquaporin redistribution				
		Stress fibres formation				
		DRA transporter inhibition				

NleH1	PP2	Pro-inflammatory				OspG
EspJ	PP2	Anti-phagocytosis				None known
NleB2	PP4	Unknown				None known
NleC	PP4	Unknown				AIP56^c^
NleD	PP4	Unknown				HopAP1, HopH1^d^
NleG	PP4	Unknown				None
NleH2	PP6	Pro-inflammatory				OspG
NleF	PP6	Unknown				None

NleA	PP6	Inhibition of protein secretion by interference	Golgi	Sec24	PDZ1	None known
(EspI)		with COPII		PDZK11		
				SNX27		
				MAlS3		
				TCOF1^b^		

NleE2	IE2	PMN transepithelial migration	Nucleus			OspZ
EspG2/Orf3	IE5	As with EspG				VirA
NleB1	IE6	Unknown				None known
NleE1	IE6	PMN trans-epithelial migration	Nucleus			OspZ
EspL2	IE6	Unknown				OspD^e^
EspL1	IE2	Pseudogene (see Figure 3)				
NleB3	IE2	Pseudogene (see Figure 3)				
EspO	PP6	Pseudogene (see Figure 3)				
Cif	PP2	Pseudogene (see Figure 3)				
NleH3	PP4	Pseudogene (see Figure 3)				

The predicted set of EPEC effectors comprises 21 full-length genes and at least 5 identified pseudogenes (i.e. genes truncated by stop codons, missing start codons, containing frameshift mutations). Where more than one copy of an effector exists, genes are numbered in accordance with sequence comparison to known EHEC homologues. For example, the full-length gene EspL is more similar to EHEC EspL2, whilst the EspL pseudogene corresponds with EspL1 and is named accordingly. All effectors are found on the chromosome in pathogenicity islands specified in [6^•^] and illustrated in Figure 3. We have attempted to include all the known and documented effector functions and the known subcellular locations from published sources. ‘Homologue family’ gives an example of a known homologue from an effector family; where indicated as ‘none known’ this does not discount similarities with unassigned hypothetical proteins. Island location is illustrated in Figure 3 in accordance with [6^•^]. All other references can be found in the text or in [3]. Phos, phosphorylation; MTS, mitochondrial targeting sequence; PRR, proline-rich repeat; SH3, src homology domains; PMN, polymorphonuclear; TJ, tight junction; IQGAP, IQ motif containing GTPase activating protein; EBP50/NHERF1, Na^+^/H^+^ exchanger regulating factor 1; ezrin–radixin–moesin, ERM-binding phosphoprotein of 50 kDa.

a We have attempted to include all the known and documented effector functions and the known subcellular locations from unpublished sources.

b ‘Functional motifs’ and ‘proposed host partners’ correspond to those that have been proved to have functional significance, although in some cases, data from yeast two-hybrid protein–protein interactions are included as in [49,50].

c *Photobacterium* virulence protein.

d *Pseudomonas syringae* effector proteins.

e Also has *Shiglella* enterotoxin homology.
